# Evaluation of two cementation protocols for lithium disilicate crowns on zirconia one-piece implants: a micro-CT analysis of cement thickness, porosity, and excess

**DOI:** 10.2340/biid.v13.45301

**Published:** 2026-01-21

**Authors:** Veranda Azizi Bunjaku, Ivica Pelivan, Rania Al-Mahdi, Blerina Azizi Veseli, Ying Xue

**Affiliations:** a School of Dental Medicine, University of Zagreb, Zagreb, Croatia; b Department of Clinical Dentistry, UiT The Arctic University of Norway, Tromsø, Norway

**Keywords:** zirconia implants, lithium disilicate crowns, micro-CT analysis, cement porosity, excess cement

## Abstract

**Objective:**

To evaluate the influence of two cementation protocols on lithium disilicate crowns cemented to zirconia one-piece implants by analyzing cement thickness, porosity, and excess cement using micro-computed tomography (micro-CT).

**Materials and methods:**

Sixteen Computer-Aided Design (CAD)/Computer-Aided Manufacturing (CAM)-fabricated lithium disilicate crowns were cemented onto zirconia one-piece implants (WhiteSKY, Bredent) using two resin-based cements: an adhesive resin cement (ARC) and a self-adhesive resin cement (SARC) based on multifunctional phosphoric methacrylates.

Each cement was applied with either a conventional apical-half (AH) or an abutment-assisted apical-half protocol (A-AH), creating four groups (*n* = 4). Samples were scanned with micro-CT for volumetric analysis of the cement. Data were analyzed using Analysis of Variance (ANOVA) with Dunnett’s post hoc test (α = 0.05).

**Results:**

The ARC showed lower porosity and more uniform cement layers than the SARC. The A-AH technique significantly reduced excess cement in both cements, particularly with the self-adhesive resin type. All groups exceeded the 50 µm digital cement space, with the self-adhesive A-AH group showing the highest thickness. Conical abutment geometry contributed to localized cement accumulations.

**Conclusions:**

Both the cement type and the application protocol appeared to influence the characteristics of the cement interface. In this study, the ARC tended to produce a more uniform and less porous cement layer, whereas the abutment-assisted protocol was associated with reduced amounts of excess cement. These findings suggest that modifications to the cementation protocol may help to optimize outcomes for zirconia one-piece implant restorations, particularly when using self-adhesive resin systems.

## Introduction

Ceramic implants have received growing interest in dentistry owing to their favorable characteristics, including biocompatibility, chemical stability, and esthetic advantages associated with their white coloration, which is particularly beneficial for patients with thin gingival biotypes seeking optimal esthetics. Clinical evidence indicates a 1-year survival rate of roughly 95% and suggests an incremental decline of approximately 0.05% per subsequent year [1, 2]. Despite these advantages, one-piece zirconia implants present challenges in cementation, as excess cement can promote biofilm retention, and their submucosal location makes thorough cleaning difficult [3–5]. Previous studies link retained cement to peri-implant disease in nearly 80% of cases, highlighting the need for removal of the cement to reduce complications and implant failure [6, 7]. In addition, the longevity of zirconia implant restorations is compromised by resin cement porosity, hydrolytic breakdown, and water sorption [8–11].

Key factors that can compromise bond strength include cement layer thickness, the presence of voids or defects, and the method of cement mixing [12]. Such porosities and voids within the cement layer can promote debonding over time and may compromise crown longevity, particularly under occlusal loading, cyclic fatigue, and exposure to the oral environment [10]. Consistent with these observations, Silva et al. reported that internal voids generated during fiber-post cementation markedly reduced push-out retention and were associated with adhesive failures at the resin–dentin interface [13]. Conversely, water sorption and hydrolytic degradation progressively compromise resin cements by cleaving the 10-Methacryloyloxydecyl dihydrogen phosphate (MDP) bond to zirconia and softening the resin matrix, thereby weakening the crown-abutment interface and promoting microleakage [14]. These flaws and hydrolytic degradation reduce retention strength and marginal integrity, increasing the risk of crown loosening or restoration failure [8, 14]. As reported by Maletin et al., cement-related factors such as porosity, hydrolytic degradation, and residual excess cement can compromise the long-term performance of resin-based luting materials, highlighting the importance of careful material selection for implant-supported restorations [15].

Lithium disilicate is popular in implant prosthodontics due to its high flexural strength in the range of approximately 360–400 MPa, translucency, and esthetics, and its reliable adhesive bonding to resin cements after proper surface treatment, making it suitable for anterior and posterior restorations [16–18]. Owing to its well-documented mechanical stability, characteristic glass-ceramic microstructure, and predictable performance under resin-cementation protocols, this material was selected for this study. These properties, together with its established use in esthetically demanding single-crown restorations, make lithium disilicate a suitable and clinically relevant substrate for evaluating cement behavior under standardized laboratory conditions.

Traditional techniques used to assess cement film quality, such as sectioning or replica methods, are often destructive and limited to two-dimensional evaluation. In contrast, micro-computed tomography (micro-CT) offers a non-destructive, three-dimensional method to visualize and quantify internal cement characteristics such as thickness, porosity, and excess cement, providing a more comprehensive evaluation of cementation outcomes [19, 20].

To the authors’ knowledge, this is the first study to employ micro-CT to simultaneously assess cement thickness, internal porosity, and excess cement in lithium disilicate crowns cemented onto zirconia one-piece implants. It is also the first to directly compare an adhesive resin cement (ARC) and a self-adhesive resin cement (SARC) using both conventional and abutment-assisted cementation techniques. These findings aim to address existing gaps in the literature and contribute to a better understanding of how cement type and protocol affect internal adaptation in implant-supported all-ceramic restorations. Accordingly, the null hypothesis was that neither the resin cement type nor the cementation protocol would significantly affect the measured cementation parameters.

## Materials and methods

### Implants and crowns

**Figure 1:**
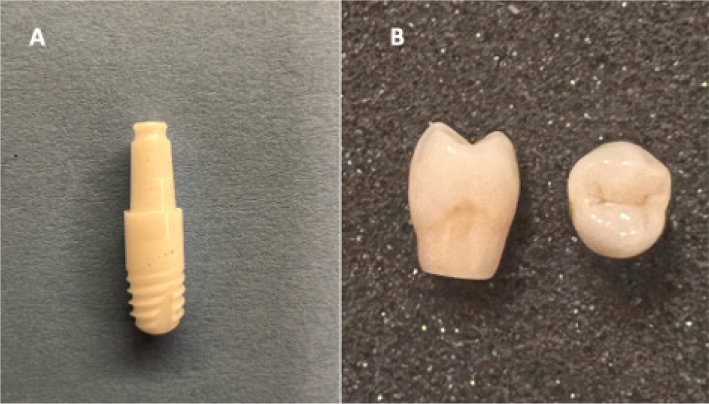
(A) One-piece zirconia Implant WhiteSky 7 at a size of 3.5 x 8mm; (B) Lithium disilicate crown (IPS e.max CAD) designed for the implant.

A total of 16 zirconia dental implants (WhiteSky 7, Bredent, Senden, Germany) and 16 lithium disilicate crowns (Ivoclar Porcelain System (IPS) e.max Computer-Aided Design (CAD), Ivoclar Vivadent, Liechtenstein) were used (Figure 1). One zirconia implant abutment was scanned using a Medit Identica Hybrid 3D dental scanner, and the scanned file was used to design and fabricate 16 standardized lithium disilicate crowns, representing a maxillary first premolar. The crowns were designed using Exocad CAD software (Exocad GmbH, Darmstadt, Germany) and milled from IPS e.max CAD blocks (shade MTA1/C14; Ivoclar Vivadent, Liechtenstein) using a PrograMill PM7 milling unit(Ivoclar Vivadent) and PrograMill Computer-Aided Manufacturing (CAM) software. The digital cement space was set to 50 µm, applied uniformly across the internal surface. Followingn milling, all restorations underwent crystallization in a Programat EP5000 furnace (Ivoclar Vivadent) according to the manufacturer’s protocol. Each crown was then characterized and glazed under identical conditions to ensure consistent surface quality. The final restorations were hand-polished with rubber polishers and a bison brush using diamond polishing paste to achieve a high-gloss finish comparable to clinically delivered crowns. The minimum crown thickness was standardized at 1.5 mm.

### Cement materials

Two different resin cements were used for the cementation procedures, i.e. Panavia V5, Kuraray Noritake Dental, Japan, an ARC, and SpeedCem Plus, Ivoclar Vivadent, Liechtenstein, a SARC. The mixing procedures and curing time are detailed in Table 1, whereas the composition and material properties of both cements are summarized in Table 2.

**Table 1:** Comparison of cement type, mixing procedure, and curing protocol for dual-cure ARC vs. the SARC.

Cement type	Dual-cure adhesive conventional group (ARC)	The self-adhesive resin cement (SARC)
**Cement name**	Panavia V5	SpeedCem Plus
**Mixing procedure**	Dispensed from auto-mix syringe; mixed automatically in the nozzle. Requires use of Tooth Primer.	Dual-paste system, auto-mix syringe. No primer or pretreatment required.
**Activating and curing time**	Touch-cure initiates polymerization upon contact with Tooth Primer. Optional tack-curing for 2–3 seconds enables gel-phase clean-up. Light-cure margins for 10–20 s. Self-cure completes in approx. 3–5 minutes.	Polymerization begins upon mixing. Optional tack-curing for 1–2 seconds enables removal of excess. Light-cure margins for 10–20 seconds if desired. Self-cure completes in approx. 2–5 minutes depending on temperature.

ARC: adhesive conventional group; SARC: self-adhesive resin cement.

**Table 2:** Reported material properties of dual-cure ARC and the self-adhesive resin cement (SARC), including chemical composition, mechanical and physical characteristics.

Property	Dual-cure adhesive conventional group (ARC)	The self-adhesive resin cement (SARC)
**Name**	Panavia V5	SpeedCem Plus
**Manufacturer**	Kuraray Noritake	Ivoclar Vivadent
**Filler loading**	61 wt% (38 vol%)	75 wt% (Base)/69.8 wt% (Catalyst)
**Base monomers**	Bis-GMA, TEGDMA, hydrophobic aromatic dimethacrylate	UDMA, TEGDMA, PEGDMA, DDDMA, MDP
**Flexural strength (MPa)**	127	≥ 60
**Flexural modulus (GPa)**	6.3	4.81
**Compressive strength (MPa)**	310	Not provided
**Film thickness (µm)**	12	33.8 ± 5.3
**Water sorption (µg/mm³)**	21	30.74 ± 2.4
**Radiopacity (% Al)**	180	400
**Fluoride release (28 days) (µg/g)**	58	Not provided
**Working time (23˚C)**	2 minutes	120–180 s
**Fillers**	Silanated barium glass filler, colloidal silica, silanated fluoroaluminosilicate glass filler	Barium glass, silica, ytterbium trifluoride

ARC group data sourced from the manufacturer Kuraray Noritake and the SARC values adapted from Ling et al. [21].

ARC: adhesive conventional group; SARC: self-adhesive resin cement; Bis-GMA: Bisphenol A-glycidyl methacrylate; UDMA: Urethane Dimethacrylate

### Cement application protocols

Each group was divided into two subgroups (*n* = 4 per subgroup) based on the cementation protocol used: Apical Half (AH) subgroup: Cement was applied to the apical half of the internal surface of the crown, and the crown was directly seated onto the implant; Abutment–Apical Half (A-AH) subgroup: Cement was applied in the same manner, but the crown was first seated on an abutment analog (index) and then removed and seated on the implant.

### Cementation procedure

**Figure 2:**
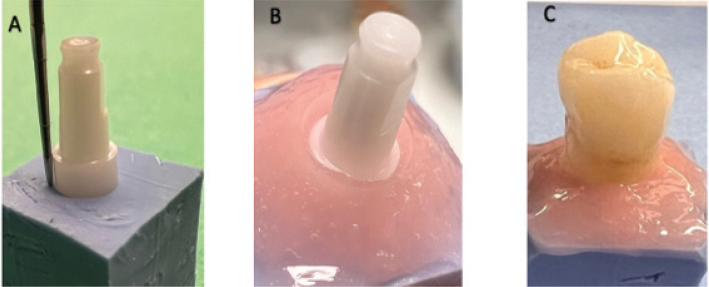
(A) Implant embedded in Aquasil putty up to the tissue-level transition; (B) Removable silicone gingival mask placed to simulate soft tissue around the implant; (C) Crown seated on the zirconia abutment prior to polymerization.

Implants were embedded in putty impression material (Aquasil Ultra+ Soft Putty, Dentsply Sirona, Germany) up to the start of the tissue-level transition (2 mm). A flexible silicone gingiva mask (UFI Gel P, VOCO, Germany) was used to simulate soft tissue (Figure 2).

The internal surfaces of the lithium disilicate crowns cemented with ARC were etched with 9% hydrofluoric acid for 20 seconds, following the Levartovsky protocol [22]. Crowns were afterward thoroughly rinsed with water for 30 seconds and gently air-dried. Clearfil™ CeramicPrimer Plus was applied using a microbrush, and subsequently air-dried without rinsing. The primer was used for both the restoration and the implant abutment within the ARC group. The crowns within the SARC group were treated with Monobond Etch&Prime.

To improve reproducibility of the cement layer, we standardized the amount of cement per crown: each coping was weighed (VWR International, Sartorius) on a precision balance (VWR International, Sartorius), 0.04 g of cement was added, and the coping was re-weighed to confirm the exact volume. This step guarantees that any differences observed in cement thickness are not attributable to variations in cement volume.

All crowns were seated using firm finger pressure by a single trained operator. Although this method was not digitally calibrated, it was applied with consistent tactile feedback to reduce variability across specimens. The approach was selected to approximate routine clinical cementation, where finger pressure is commonly employed. Excess cement was removed after tack curing using a carbon fiber implant scaler. A subsequent assessment was done after complete polymerization to determine if additional removal of cement was necessary.

### Micro-CT analysis

Each sample was scanned using a micro-CT scanner (SkyScan 1272; Bruker-microCT, Kontich, Belgium) at 100 kV and 100 μA with a 0.11 mm copper filter. All scans were performed at a resolution of 15 μm using 360-degree rotation, random movement of 20 mm, three-frame averaging with a camera exposure time of 1400 ms, and a rotation step of 0.4 degrees. Data were reconstructed using a ring artefact reduction factor of 6 and a beam hardening correction of 20% with NRecon v. 1.6.9.18 software (Bruker-microCT). The CTAn v.1.14.4.1 software (Bruker microCT) was used for quantification for volumetric and structural analysis of cement thickness, porosity, and excess material.

Using CTAn software, the cement layer was segmented from the dataset, and internal voids were identified by a lower density threshold; total void volume (mm^3^) and percent porosity (void volume as a percentage of total cement volume) were calculated for each sample.

Excess cement was defined as cement material extending beyond the crown margin, and its volume was measured separately. Cement layer thickness was measured on micro-CT cross-sectional images using ImageJ (National Institutes of Health, Bethesda, MD, USA). Measurements were performed at five standardized locations per specimen along the crown–abutment interface. The mean thickness per specimen was calculated from these five values. Calibration and scale setting were based on voxel dimensions obtained from the micro-CT scan parameters.

### Statistical analysis

Data were analyzed using GraphPad Prism (version 10.3.1). Normality was assessed using the Shapiro–Wilk test, and homogeneity of variances was evaluated using Bartlett’s test. Group comparisons were performed using one-way Analysis of Variance (ANOVA) followed by Dunnett’s post hoc test. Results are presented as mean ± standard deviation, and a *p*-value < 0.05 was considered statistically significant. G*Power analysis was performed to estimate the minimum required sample size for the evaluated outcomes, and a posteriori power analysis was performed in Statistica after the ANOVA tests.

## Results

### Porosity and voids analysis

**Figure 3:**
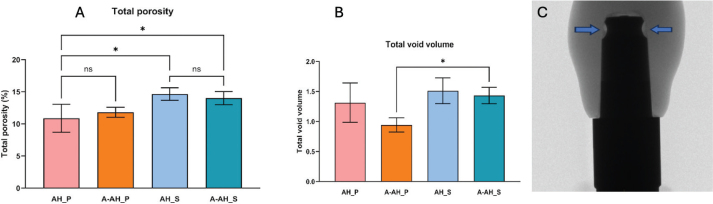
Micro-CT-based analysis of cement porosity and voids across different cementation protocols. (A) Total porosity (%) in four groups. Significant differences were observed between AH_S and AH_P and between A-AH_S and AH_P (*p* < 0.05); (B) Total void volume (mm³) among groups, with a significant difference observed between A-AH_S and A-AH_P (*p* < 0.05). Bars indicate mean ± Standard Deviation (SD); (C) Representative micro-CT scan showing the cement interface between crown and abutment, highlighting localized voids. CT: computed tomography.

Micro-CT analysis revealed differences in both porosity percentage and total void volume among the four groups. In terms of porosity (%), SARC AH_S exhibited the highest internal porosity (14.01 ± 2.0%), followed closely by SARC A-AH_S (14.65 ± 2.0%). The ARC AH_P showed the lowest porosity (10.88 ± 2.0%), while the ARC A-AH_P measured 11.83 ± 2.0% (Figure 3). Statistically significant differences were found between AH_P and AH_S (*p* = 0.0122), and between AH_P and A-AH_S (*p* = 0.0406); no other comparisons reached significance (*p* > 0.05).

Regarding total void volume (mm³), the highest mean value was observed in SARC AH_S (1.514 ± 0.22 mm³), followed by A-AH_S (1.433 ± 0.22 mm³) and AH_P (1.314 ± 0.22 mm³). The A-AH_P exhibited the lowest void volume (0.944 ± 0.22 mm³) (Figure 3). Statistically significant differences were observed between A-AH_P and AH_S (*p* = 0.0442), and between A-AH_P and A-AH_S (*p* = 0.0169). These findings suggest that both the cement type and cementation technique influence internal porosity characteristics, where the ARC A-AH_P demonstrates the most favorable internal adaptation.

### Excess cement analysis

**Figure 4:**
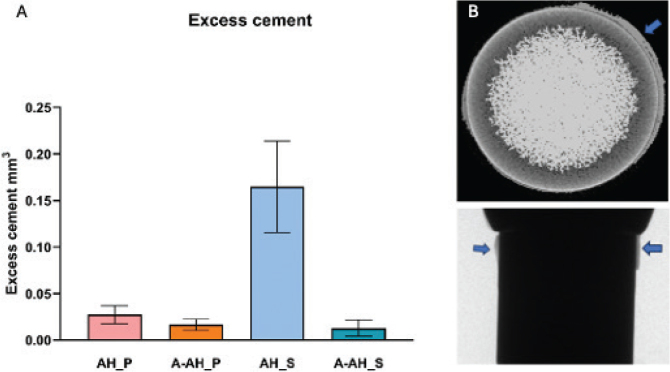
Excess cement analysis. (A) Excess cement volume; significantly higher in the conventional self-adhesive resin cement (SARC) (*p* < 0.0001) group (*p* < 0.05). Bars show mean ± SD; (B) Representative micro-CT scan showing cement extrusion beyond the crown margin. CT: computed tomography.

Analysis of cement extrusion beyond the crown margins revealed noticeable variation among the tested groups. Specimens in the SARC AH_S subgroup using conventional application demonstrated the greatest amount of excess cement (0.1648 ± 0.026 mm³), which was significantly greater than that of all other subgroups (*p <* 0.0001), whereas the SARC A-AH_S exhibited significantly lower overflow with a mean of 0.013 ± 0.026 mm³ (*p <* 0.0001 vs. AH_S). In contrast, both ARC AH_P and A-AH_P showed minimal cement extrusion regardless of the application protocol. The AH_P measured 0.027 ± 0.026 mm³, and the A-AH_P measured 0.017 ± 0.026 mm³, with no significant difference between them (*p* = 0.9316) (Figure 4).

These findings indicate that the incorporation of an abutment analog enhances cement control, particularly with low-viscosity self-adhesive materials. Controlling excess cement is critical for peri-implant tissue preservation and may reduce the risk of inflammation associated with subgingival cement remnants.

### Cement thickness analysis

**Figure 5:**
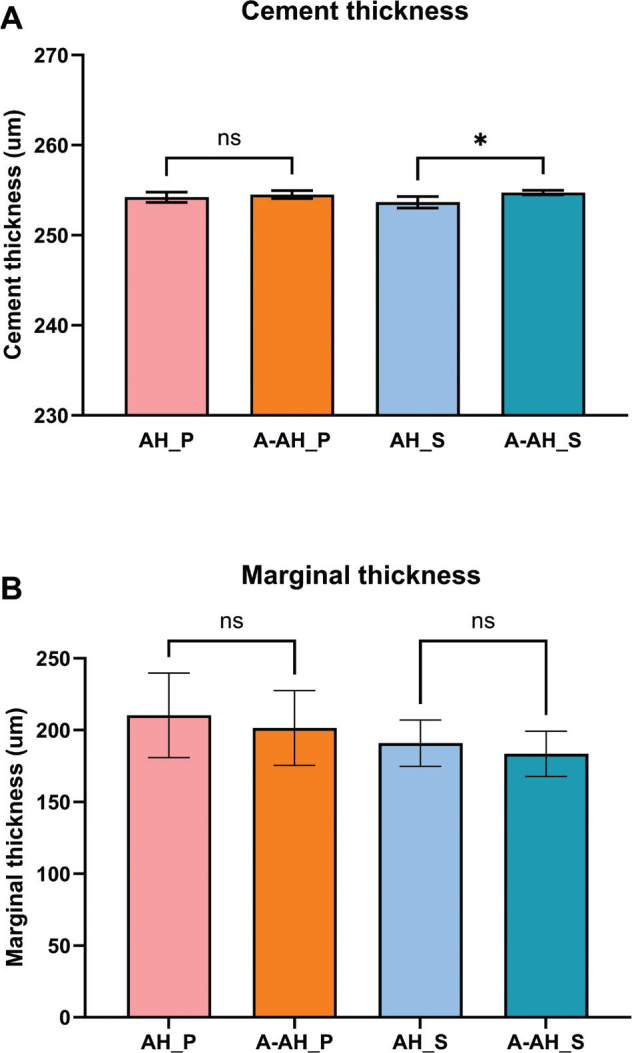
A. Mean cement thickness (μm) for each group. A-AH_S exhibited significantly greater thickness than AH_S (p = 0.0467). B. Cement thickness in the marginal area. No statistically significant difference was detected Bars represent standard deviation.

All specimens showed continuous cement layers at the crown–implant interface, with minor differences in thickness among the groups. The SARC AH_S subgroup had the lowest mean cement thickness (253.7 ± 0.5 µm), while the SARC A-AH_S subgroup had the highest (254.7 ± 0.5 µm). This difference was statistically significant (*p* = 0.0467) (Figure 5). The ARC AH_P and A-AH_P subgroups showed intermediate and consistent values: AH_P measured 254.2 ± 0.5 µm, and A-AH_P measured 254.5 ± 0.5 µm, with no significant difference between them (*p* > 0.05). Despite the statistically significant difference between the self-adhesive subgroups, all measured cement thicknesses remained within clinically acceptable limits. The marginal cement thickness values were comparable across all groups. Statistical analysis revealed no significant differences among any of the groups (*p* > 0.05) for marginal cement thickness.

## Discussion

The null hypothesis for this study was partially rejected, as both the resin cement type and the cementation protocol significantly influenced the cementation outcomes. An ARC and a SARC were selected for this study to investigate how differing adhesive strategies influence cementation outcomes in implant-supported restorations. These two cements were chosen to represent two different bonding mechanisms and material behaviours. The principal distinction between the two cements lies in the location of the MDP monomer; ARC relies on an extra-oral primer that contains MDP [23], whereas SARC embeds MDP within the cement matrix, eliminating the primer step but delivering the monomer at a lower interfacial concentration [21]. This formulation difference is accompanied by divergent material properties: ARC has a lower filler loading, higher proportion of rigid Bis-GMA, thin film thickness and brief light-curing time favor rapid polymerization and stronger chemical adhesion, whereas SARCs higher filler content, flexible UDMA-based monomers, and thicker film result in longer working/curing times and lower flexural strength (Table 2). Under the conditions tested, ARC showed lower porosity values and fewer internal voids, particularly in combination with the A-AH technique, relative to the SARC. The observed differences in internal porosity can be explained by the cements’ composition and curing characteristics.

SARC have been reported to present a slower rate of polymerization and a lower final degree of conversion than conventional resin cements, in either the dual- or self-cure mode [24]. Frassetto et al. further showed that, in self-adhesive systems, the maximum degree of conversion was reached earlier than the peak contraction stress, indicating a period after light activation during which the material continues to develop stress while still undergoing structural changes [10]. Such a prolonged pre-gel or gelation phase could, in theory, provide more opportunity for entrapped air to persist within the cement mass before final hardening. In the same study, the conventional resin-based cement exhibited higher contraction stress than the self-adhesive cements, which is consistent with a different stress-development pattern [10]. Although the materials evaluated in these investigations are not identical to those used in this study, the general trends described in the literature may offer a plausible explanation for the higher porosity measured in the self-adhesive group relative to the adhesive dual-cure system.

Zeller et al. reported that certain adhesive dual-cure resin cements exhibited higher initial flowability followed by a rapid increase in viscosity during the early stages of polymerization, whereas specific counterparts, self-adhesive cements investigated in the same study demonstrated a comparatively higher initial viscosity under identical conditions [25]. These findings suggest that adhesive dual-cure systems may show a more favorable flow pattern during seating pressure, whereas Ling et al. found that the SARC they evaluated contained a relatively high filler content (75 wt% in the base paste), which may influence its handling and flow characteristics [21]. Although neither study examined the exact cements used in our investigation, the established principle that elevated filler loading restricts polymer chain mobility and increases viscosity provides a plausible explanatory framework. We hypothesize that the comparatively reduced flow observed for our self-adhesive cement, and the associated increase in internal void formation, may reflect similar compositional characteristics – particularly high filler volume fraction limiting initial seating adaptation. This interpretation remains speculative, given the material formulation differences, and warrants direct rheometric validation.

Despite the differences between groups, the overall porosity values remained low in this study. This may be partly related to the use of automix delivery systems, which minimize operator-dependent mixing variability. Sadighpour et al. reported that differences in mixing methods can lead to differences in porosity and that auto-mix formulations may show improved integrity compared with manually mixed versions [8]. In addition to porosity, total void volume was also quantified to provide complementary information about internal defects within the cement layer. Because only total void volume was quantified, this study does not provide information on void distribution or morphology. To our knowledge, no previous micro-CT investigation has directly compared void formation between SARC and ARC in crown cementation, and existing reports have focused on other clinical models or on single cement types. Within these constraints, the higher total void volume observed in the SARC groups may reflect differences in viscosity or polymerization behaviour reported in the literature for these materials; however, this interpretation should be considered preliminary and would require confirmation through more detailed micro-CT evaluations. A discrepancy was also observed between percentage porosity and total void volume: the ARC group exhibited lower overall percentage porosity but fewer, larger voids, whereas the SARC group showed a greater number of smaller defects. This pattern reflects the distinct meaning of each variable: percentage porosity represents the fraction of voids relative to the entire cement volume, whereas total void volume describes the absolute void space, a distinction also highlighted in previous micro-CT assessments of resin cements and posts [26, 27].

Cement layer thickness exceeded the digitally prescribed 50 µm in all groups, with mean values surpassing 250 µm. Comparable discrepancies have been reported in the literature; for example, Sampaio et al. found that CAD/CAM crowns fabricated with a 120 µm die-spacer exhibited an actual mean cement thickness of approximately 240 µm, about twice the intended value [28]. These findings indicate that digitally programmed spacer settings may not precisely predict the final cement film thickness obtained during CAD/CAM fabrication. In this study, it is plausible that the conical geometry of the implant abutments contributed to localized increases in cement thickness due to variations in cement flow during seating. Although this remains a hypothesis based on our observations, previous studies have demonstrated that abutment geometry significantly affects cement retention and cement distribution patterns [29, 30]. The influence of cement film thickness on crown retention is well established. Mehl et al. reported that increasing the film thickness from 15 to 50 µm was associated with a measurable reduction in retention strength (*p* ≤ 0.001) [31], regardless of the cement used. These results suggest that even relatively small variations in film thickness may influence the retentive behavior of cemented restorations, highlighting the relevance of precise crown seating and controlled cement application. Cement film thickness is a multifactorial property, and consistency – reflecting the flowability and handling characteristics of the material – is one of its key determinants. Consistency is influenced by factors such as filler content, resin composition, and early polymerization behavior, which collectively affect how the material spreads under seating pressure.

Cements with higher filler loading or more viscous monomer matrices typically exhibit reduced flowability, limiting their capacity to form thin and uniform films during crown seating [21]. In our study, SARC exhibited thicker layers than ARC, a pattern consistent with literature reporting that self-adhesive cements generally display higher viscosity and reduced flow due to their formulation [21]. Taken together, these observations indicate that both intrinsic material properties and the cementation protocol contribute to the final cement film thickness and its distribution. Marginal cement thickness, i.e. the cement layer measured at the crown-implant margin, behaved differently. Statistical analysis revealed no significant differences (*p* > 0.05) (Figure 5).

Given the non-retrievable design of one-piece zirconia implants, preventing cement overflow is critical to long-term biological success. Our abutment-assisted approach was adapted from the extraoral strategy reported by Jagathpal et al. While their method involves seating the crown on a duplicate abutment, and ours uses the actual implant abutment, both techniques share the same rationale: an initial extraoral seating step to extrude and remove excess cement before definitive placement [32]. Excess cement volume was lower when the A-AH protocol was applied, with a more noticeable reduction in the SARC group. These results indicate that the abutment-assisted approach can help to limit cement extrusion. Comparable trends have been reported in studies evaluating modified cementation techniques aimed at reducing marginal overflow [33, 34]. Our findings are in line with these observations and suggest that the A-AH protocol may be a useful strategy for controlling excess cement in implant restorations. Notably, ARC demonstrated low excess cement volumes in both application protocols. This may be related to its lower viscosity and handling characteristics, which could permit more controlled seating and facilitate removal during the tack-curing phase [23]. In contrast, SARC, which has a higher viscosity, showed greater cement extrusion when applied conventionally. This observation suggests that technique modification may be particularly relevant when working with more viscous self-adhesive materials.

A previous study by Gønder et al. investigated extraoral replicas and vented abutment designs and reported that these approaches helped reduce residual cement; the authors concluded that both cement type and cementation technique should be considered when aiming to limit excess cement [33]. While this study did not incorporate vented abutments or replica-based procedures, the abutment-assisted protocol used here includes an extraoral component with a similar objective of improving cement control. In the current findings, this protocol was associated with reduced overflow in the SARC, which aligns with the general principle described by Gønder et al. However, the ARC exhibited low excess cement independent of protocol, indicating that intrinsic material properties also influenced the outcomes. Overall, the results partially correspond with those of Gønder et al., while also reflecting methodological and material-related differences between the studies.

The limited specimen number is an important limitation of this study. The G*Power analysis indicated that six specimens per group would be required for porosity and cement-thickness measurements, whereas fewer samples would be sufficient for void-volume and excess-cement outcomes. Although four specimens were included per group, the observed post hoc power calculated in Statistica exceeded 0.80 for all ANOVA tests. Power considerations are most relevant when interpreting non-significant findings due to the risk of Type II error, and future studies with larger sample sizes would allow a more comprehensive assessment of group differences. This study examined two resin cements; the results may not generalize to all available cements. However, the trends observed here (e.g. differences in porosity and excess cement between an adhesive vs. self-adhesive system) provide insight that can likely extend to similar materials with comparable properties. Future studies, including larger sample sizes and a broader range of cements, would be valuable to confirm these observations.

## Conclusion

Within the limitations of this in vitro study – particularly the small sample size due to the resource-intensive nature of micro-CT analysis – the results indicate that both the resin cement type and the cementation technique can influence factors such as cement thickness, porosity, and excess cement. The abutment-assisted protocol was associated with reduced cement overflow, particularly in the self-adhesive cement group. The ARC AH_P showed comparatively lower porosity and more uniform adaptation under the conditions tested. These findings highlight the relevance of considering material-specific properties and application techniques when selecting cementation protocols for one-piece zirconia implant restorations. Further clinical research involving larger sample sizes and a broader range of cement types is needed to assess the generalizability and clinical implications of these results.
